# Hepatic Relaxation Times from Postmortem MR Imaging of Adult Humans

**DOI:** 10.2463/mrms.mp.2015-0086

**Published:** 2015-12-22

**Authors:** Seiji SHIOTANI, Tomoya KOBAYASHI, Hideyuki HAYAKAWA, Kazuhiro HOMMA, Harumi SAKAHARA

**Affiliations:** 1 Department of Radiology, Seirei Fuji Hospital 3-1 Minami-cho, Fuji, Shizuoka 417-0026, Japan; 2 Department of Radiological Technology, Tsukuba Medical Center Hospital; 3 Department of Forensic Medicine, Tsukuba Medical Examiner’s Office; 4 Evaluation Department, National Institute of Advanced Industrial Science and Technology; 5 Department of Radiology, Hamamatsu University School of Medicine

**Keywords:** liver, in vivo, low body temperature, T_1_ and T_2_ values, postmortem magnetic resonance imaging

## Abstract

**Purpose:**

To measure T_1_ and T_2_ values of hepatic postmortem magnetic resonance (PMMR) imaging.

**Materials and Methods:**

We performed hepatic PMMR imaging of 22 deceased adults (16 men, 6 women; mean age, 56.3 years) whose deaths were for reasons other than liver injury or disease at a mean of 27.7 hours after death. Before imaging, the bodies were kept in cold storage at 4°C (mean rectal temperature, 17.6°C). We measured T_1_ and T_2_ values in the liver at two sites (the anterior segment of the right lobe and the lateral segment of the left lobe). We also investigated the influence of the body temperature and postmortem interval on T_1_ and T_2_ values.

**Results:**

In the anterior segment of the right lobe and the lateral segment of the left lobe, T_1_ values of PMMR imaging were 524 ± 112 ms and 472 ± 104 ms (mean ± standard deviation), respectively; while T_2_ values were 42 ± 6 ms and 43 ± 8 ms, respectively. T_1_ and T_2_ values did not differ significantly between the two sites (*P* ≧ 0.05). Regarding temperature, the T_2_ values of hepatic PMMR imaging were linearly correlated with the body temperature, but the T_1_ values were not. The T_1_ and T_2_ values of the two sites in the liver did not correlate with the postmortem interval.

**Conclusion:**

Reduction in body temperature after death is considered to induce T_1_ and T_2_ value changes in the liver on PMMR imaging.

## Introduction

While the worldwide decline in the rate of conventional autopsies has increased the need for and frequency of postmortem imaging as a complementary, supplementary, or alternative method of autopsy,^1–8^ there continues to be insufficient postmortem imaging data for some parts of the human body due to lack of data obtained at various imaging settings.

Postmortem magnetic resonance (PMMR) imaging can provide more detailed information with better contrast resolution than postmortem computed tomography (PMCT), and depict some pathological conditions that are difficult to identify with PMCT.^9^ However, PMMR imaging has revealed that both MR signals and image contrast change after death, which can deteriorate diagnostic accuracy.^9–13^ The optimization of parameters for PMMR imaging and accurate interpretation of imaging findings require analyses of quantitative data.^14–16^ T_1_ and T_2_ values on MR imaging of the human adult liver *in vitro* and *in vivo* have been reported.^17–22^ We surmised that the quantitative data of T_1_ and T_2_ values of hepatic PMMR imaging of *in vivo* would help interpret postmortem imaging variation relative to postmortem interval (time elapsed after death), functional failure before death, and/or metabolic abnormality resulting from pharmacologic or toxic substances. A newly published first-impression postmortem study using a 3.0T scanner reported that different magnetic field strengths, such as 1.5T, would result in different quantitative values for T_1_ and T_2_ for the same tissue and temperature.^16^ We report the T_1_ and T_2_ values of 1.5T PMMR imaging of an adult human liver *in vivo*.

## Materials and Methods

### Subjects

We examined PMMR imaging data of 22 adults (16 men, 6 women; aged 27–83 years, mean: 56.3 years) who died suddenly and unexpectedly and did not have abnormal results nor indications of fatty liver on hepatopathological examination. Both ascites around the liver and putrefaction gas formation were ruled out using whole-body PMCT immediately before PMMR imaging. Their bodies were kept in cold storage at 4°C and subjected to PMMR imaging 7 to 96 hours after the confirmation of death (mean: 27.7 hours). Their rectal temperatures, measured immediately after PMMR imaging with an industrial thermometer (7-257-01, AS ONE Corp., Osaka, Japan), were 5–31°C (mean: 17.6°C).

Autopsy was performed on each subject after PMMR imaging. Causes of death were 5 cases of trauma not involving the abdomen (cervical injuries due to road traffic accidents), 5 cases of ischemic heart disease (coronary arterial thrombus and/or myocardial infarction), 5 cases of acute heart failure due to fatal arrhythmia, 3 cases of drowning in the bathtub, 2 cases of cerebral hemorrhage, and 1 case each of subarachnoid hemorrhage and acute drug intoxication.

### Scan conditions

With the permission of our institutional ethics committee, we performed PMMR imaging using a 1.5T MR imaging clinical scanner (Avanto, Siemens, Erlangen, Germany) with a dedicated 6-channel body matrix coil and spine matrix coil. We measured T_1_ and T_2_ values with a relaxation time map creation tool (syngo MapIt, Siemens, Erlangen, Germany).^23^ Table 1 shows the scan parameters for the liver. A dual-flip angle technique based on the 3D-FLASH spoiled gradient echo sequence was employed for T_1_ mapping. T_2_ mapping was based on a multi-echo spin echo sequence. However, the first echo was ignored in the pixel-wise calculation of the T_2_ map, since it consistently yielded a lower signal than the second echo. Inhomogeneity correction was performed for static magnetic field (B_0_); however, it was not performed for radiofrequency magnetic field (B_1_).

### Analyses

**Fig. 1.**
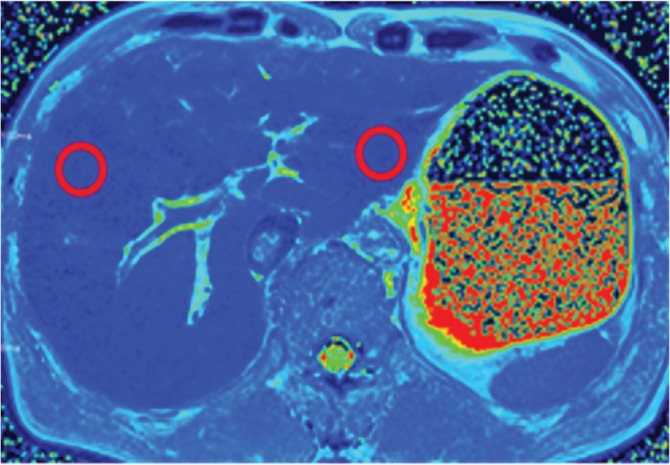
Postmortem 1.5T magnetic resonance (MR) image of the liver. Red circles indicate regions of interest (ROIs) placed at the level of the main trunk of the portal vein on postmortem MR images. ROIs were placed on the anterior segment of the right lobe and lateral segment of the left lobe.

A radiological technologist (T.K.) with 16 years of experience defined 2-cm-diameter circular regions of interest (ROIs) in the liver in both the anterior segment of the right lobe and the lateral segment of the left lobe excluding the vessels and bile ducts on axial images at the level of the main trunk of the portal vein (Fig. 1).

Statistical analyses were performed using statistical software (Excel 2010, Microsoft, Redmond, WA, USA) with Statcel 2 (OMS, Tokyo, Japan) an add-in software. Parametric statistics (arithmetic mean value ± standard deviation [SD]) and Student’s t-test were used with a significance value of *P* < 0.05 for group differences.

The relationships among T_1_ values, T_2_ values, and rectal temperatures were analyzed with Pearson’s correlation coefficient using the least squares methods. Also, the relationships among T_1_ values, T_2_ values, and postmortem intervals were analyzed using the same methods.

## Results

**Fig. 2.**
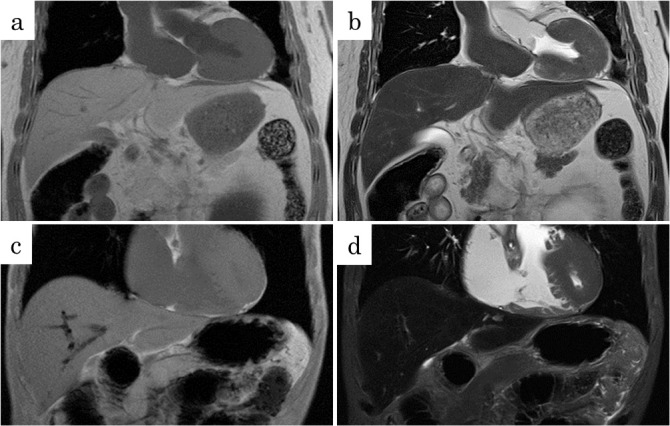
(**a**) Coronal T_1_-weighted image of the liver on postmortem magnetic resonance imaging (MRI). T_1_ value of the anterior segment of the right lobe and lateral segment of the left lobe is 474.3 and 571.9 ms (rectal temperature: 27.5°C). (**b**) Coronal T_2_-weighted image of the liver on postmortem MRI. T_1_ value of the anterior segment of the right lobe and lateral segment of the left lobe is 57.7 and 54.0 ms (rectal temperature: 27.5°C). (**c**) Coronal T_1_-weighted image of the liver on postmortem MRI. T_2_ value of the anterior segment of the right lobe and lateral segment of the left lobe is 500.5 and 401.5 ms (rectal temperature: 5.0°C). (**d**) Coronal T_2_-weighted image of the liver on postmortem MRI. T_2_ value of the anterior segment of the right lobe and lateral segment of the left lobe is 29.9 and 26.8 ms (rectal temperature: 5.0°C).

A diagnostic radiologist (S.S.) with 24 years of experience observed no specific abnormality, except for signal intensity and contrast, between PMMR imaging (Fig. 2a–d) and clinical MR imaging of the liver. The T_1_ values of the anterior segment of the right lobe and the lateral segment of the left lobe were 524 ± 112 ms and 472 ± 104 ms (means ± SD), respectively (Table 2), showing no significant difference between the two sites (*P* = 0.123, t = 1.575 < 2.018 = t_0.05(42)_). The T_2_ values of the anterior segment of the right lobe and the lateral segment of the left lobe were 42 ± 6 ms and 43 ± 8 ms, respectively (Table 2), showing no significant difference between the two sites (*P* = 0.823, t = 0.225 < 2.018 = t_0.05(42)_).

**Fig. 3.**
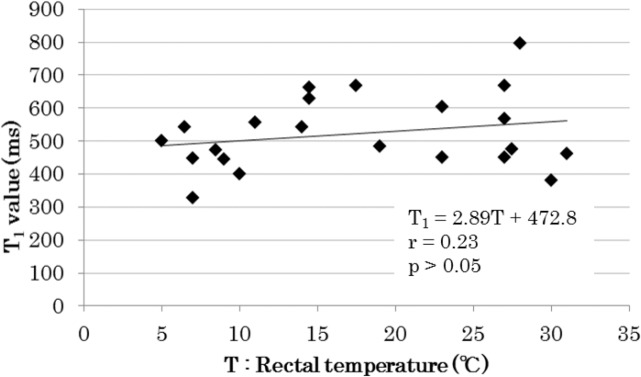
Relationship between T_1_ values of the anterior segment of the right lobe and rectal temperature of deceased subjects.

**Fig. 4.**
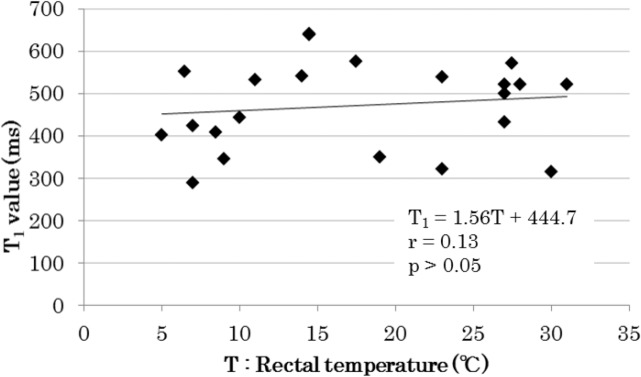
Relationship between T_1_ values of the lateral segment of the left lobe and rectal temperature of deceased subjects.

**Fig. 5.**
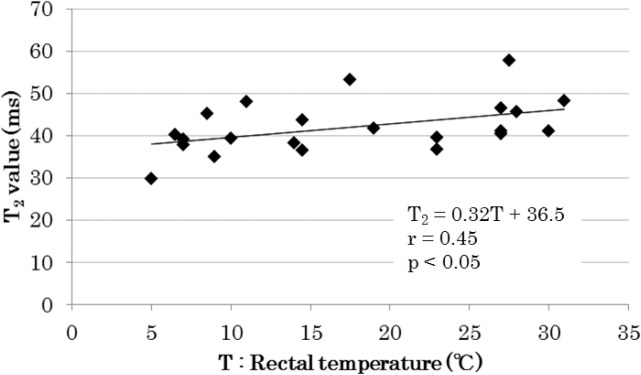
Relationship between T_2_ values of the anterior segment of the right lobe and rectal temperature of deceased subjects.

**Fig. 6.**
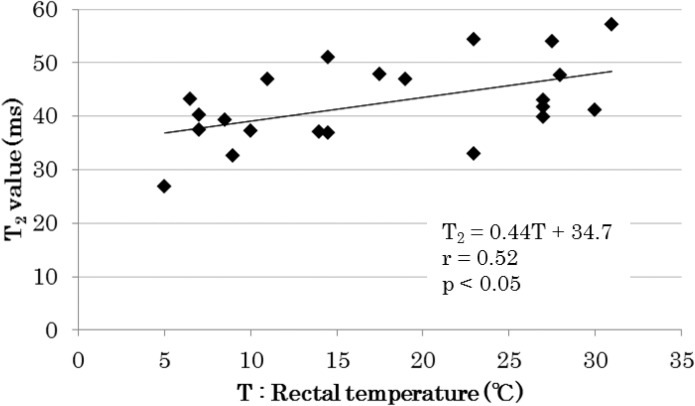
Relationship between T_2_ values of the lateral segment of the left lobe and rectal temperature of deceased subjects.

Regarding rectal temperature, with hepatic PMMR imaging, T_1_ values of the anterior segment of the right lobe did not correlate significantly with the temperature (correlation coefficient r = 0.23; *P* > 0.05, Fig. 3); nor did T_1_ values of the lateral segment of the left lobe correlate significantly with the temperatures (r = 0.13; *P* > 0.05, Fig. 4). In contrast, T_2_ values of the anterior segment of the right lobe (r = 0.45; *P* < 0.05, Fig. 5) and T_2_ values of the lateral segment of the left lobe (r = 0.52; *P* < 0.05, Fig. 6) significantly correlated with the rectal temperature.

**Fig. 7.**
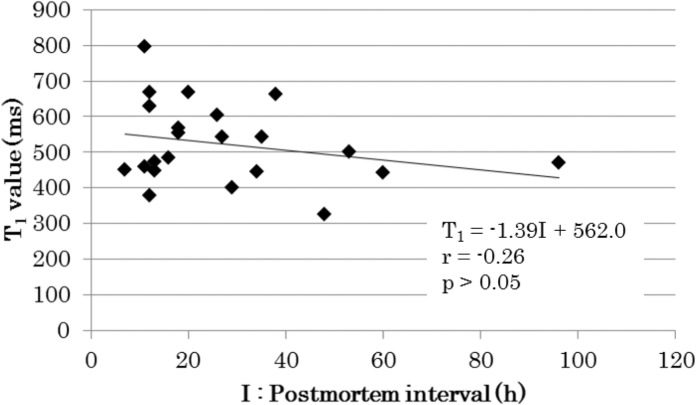
Relationship between T_1_ values of the anterior segment of the right lobe and postmortem interval of deceased subjects.

**Fig. 8.**
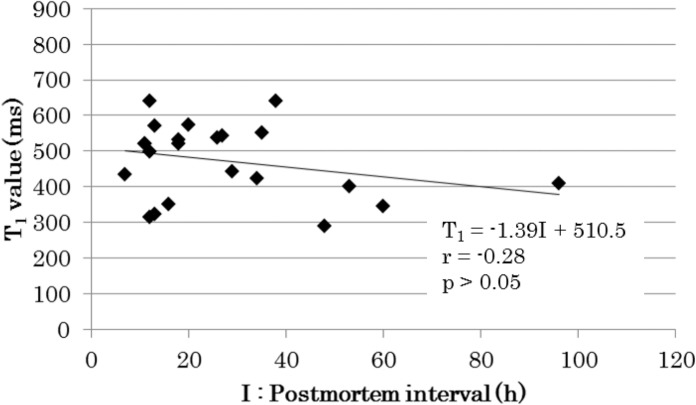
Relationship between T_1_ values of the lateral segment of the left lobe and postmortem interval of deceased subjects.

**Fig. 9.**
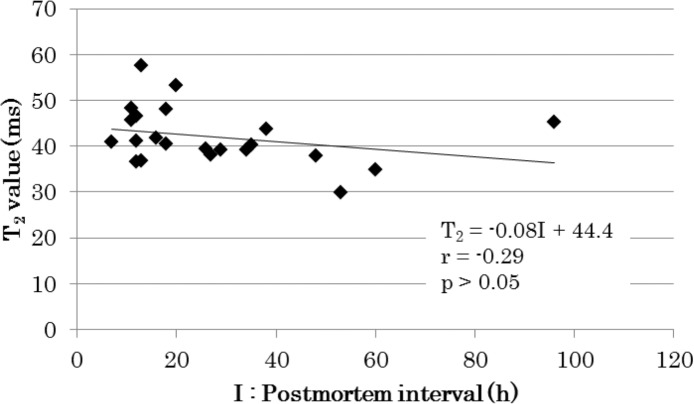
Relationship between T_2_ values of the anterior segment of the right lobe and postmortem interval of deceased subjects.

**Fig. 10.**
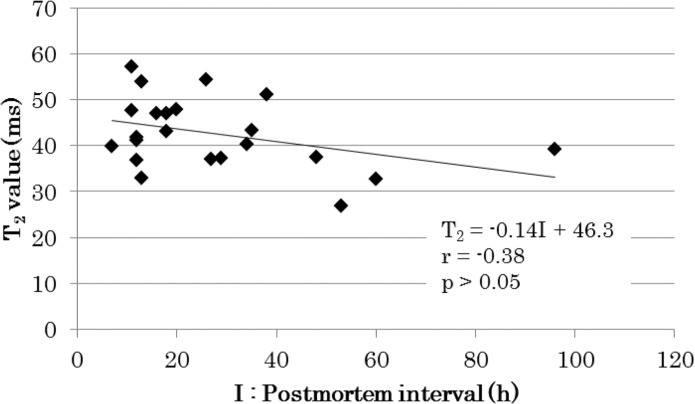
Relationship between T_2_ values of the lateral segment of the left lobe and postmortem interval of deceased subjects.

Regarding postmortem interval, with hepatic PMMR imaging, T_1_ values of the anterior segment of the right lobe (correlation coefficient r = −0.26; *P* > 0.05, Fig. 7) and T_1_ values of the lateral segment of the left lobe (r = −0.28; *P* > 0.05, Fig. 8) did not correlate significantly with the postmortem interval. Also, T_2_ values of the anterior segment of the right lobe (r = −0.29; *P* > 0.05, Fig. 9) and T_2_ values of the lateral segment of the left lobe (r = −0.38; *P* > 0.05, Fig. 10) did not correlate significantly with the postmortem interval.

## Discussion

In the present study, the average T_1_ values of the anterior segment of the right lobe and the lateral segment of the left lobe on hepatic PMMR imaging at 1.5T were 524 ± 112 ms and 472 ± 104 ms, respectively. The average T_2_ values of those two sites were 42 ± 6 ms and 43 ± 8 ms, respectively. T_2_ values of the two sites linearly correlated with the body temperature, but T_1_ values did not. T_1_ and T_2_ values of the two sites did not correlate with the postmortem interval.

Zech et al. reported T_1_ and T_2_ values of hepatic PMMR imaging at 3.0T were 680 and 37 ms.^16^ De Bazelaire et al. reported that T_1_ values were generally higher and T_2_ values were generally lower at 3.0T than at 1.5T in the clinical MR imaging,^22^ which agree with the results of our study and those of Zech et al. In the report of de Bazelaire et al., four healthy adult volunteers underwent MR imaging with a whole-body 1.5T MR scanner with a receive-only surface coil array, and T_1_ and T_2_ values of the liver were measured using an inversion-recovery method with different inversion times and a multiple spin-echo technique with different echo times.^22^ Average T_1_ and T_2_ values of their living subjects’ livers were 586 ± 39 ms and 46 ± 6 ms, respectively. Comparing measured T_1_ and T_2_ values between our deceased subjects and living subjects in the report of de Bazelaire et al.,^22^ hepatic PMMR imaging showed a significantly shorter T_1_ value of the lateral segment of the left lobe, but no significant differences were noted in the anterior segment of the right lobe and T_2_ values of the both sites.

In the liver after death, decomposition and cooling^24^ affects T_1_ and T_2_ values of MR imaging due to changes in the components and property of the liver, including fat, water, and paramagnetic substances such as iron,^25^ all of which are expressed as relaxation time changes of the entire liver. In a living body, motion-induced imaging artifacts occur due to pulsatile blood flow; thus, deviations in the measurement of T_1_ and T_2_ values are normalized. However, with PMMRI, T_1_ and T_2_ values do not include such artifacts.

As decomposition occurs after death, the increase of phosphoric, carbonic, fatty, and lactic acids induces acidosis of the liver and reduces the pH.^26,27^ Investigating the livers of rats, Moser et al. reported that reduction of the pH caused the prolongation of T_2_ values, though they did not mention T_1_ values.^28^ We suggest that pH reduction in the postmortem human liver can cause the prolongation of T_2_ values.

The increased water content of tissues causes the elevation of T_1_ and T_2_ values,^29^ although those of the liver are not the focus of attention in our study. Following death, the brain shows a time-course increase in water content due to the absorption of spinal fluid around the brain.^30,31^ This is attributed to increased lactic acid as a result of anaerobic glycolysis and increased osmotic pressure due to an increased number of proteins caused by autolysis in the ischemic brain.^32–34^ However, the liver in our study is considered not to have shown increased water content for a similar reason, because none of our cases exhibited ascites around the liver. An increase in the water content of the liver is known to sometimes occur with a prolonged agonal stage,^35^ although this does not apply to our study since we investigated sudden death cases.

The Bloembergen-Purcell-Pound theory states that changes in T_1_ and T_2_ values are related to temperature change.^36^ Therefore, the cooling of the body after death and subsequent storage of the cadaver in a refrigerator are considered to be the causes of MR relaxation time changes. Within a small temperature range, the T_1_ value depends linearly on the temperature in fat and water.^14,15,37–41^ The T_2_ value decreases with a declining temperature in fat tissue and aqueous solutions;^37,40,41^ however, the temperature dependence of the T_2_ value can be masked by other factors in tissue.^37^

The magnetic susceptibility of a paramagnetic substance is inversely proportional to the absolute temperature according to Curie’s law (magnetic susceptibility χ = C/T, where C is a constant and T is the absolute temperature).^37^ The liver contains some paramagnetic substances such as iron, manganese, and copper.^42–44^ The increased magnetic susceptibility associated with temperature reduction induces changes in the relaxation time and has a very weak T_1_-shortening effect but strong T_2_-shortening effect.^45^

In the liver of our PMMR imaging, T_1_ values were shorter than the reported clinical MR imaging^22^ in the lateral segment of the left lobe, but not much difference was found in the anterior segment of the right lobe. This T_1_-shortening effect is due to the effect of fat and water at low temperatures. However, T_1_ values on hepatic PMMR imaging did not correlate significantly with the rectal temperature. Matsumoto et al.^38^ reported that the T_1_ value of a pig liver *in vitro* decreased linearly with a temperature reduction range of 10–50°C, although the T_1_ value increased between the range of 0–10°C. Moser et al.^28^ reported that in a temperature below 37°C, at 4 hours after biopsy excision, T_1_ values were higher in a rat liver *in vitro* at 22, 15, 30, 7, and 37°C, in this order. Namely, T_1_ value reduction was not proportional to the temperature reduction. In our study, the T_1_ value of the postmortem liver varied at low temperature over a similar range, which is considered to be the reason for not showing linearity regarding the relationship between the T_1_ value and rectal temperature.

Regarding T_2_ values, compared with clinical MR imaging of the liver, hepatic PMMR imaging did not show any significant change. For the liver, T_2_ prolongation due to a reduced pH, T_2_ shortening due to fat, water, and paramagnetic substances at low temperature negated each other. However, T_2_ values on hepatic PMMR imaging were correlated significantly with the rectal temperature. Also, according to Moser et al., at 4 hours after biopsy excision in a temperature below 37°C, high T_2_ values of a rat liver *in vitro* were observed at 37, 30, 22, 15, and 7°C in this decreasing order, showing the proportional reduction of T_2_ values relative to the temperature. In our study, the relationship between T_2_ value and rectal temperature of the liver showed linearity.

With hepatic PMMR imaging, T_1_ values did not correlate significantly with the rectal temperature, but T_2_ values significantly correlated. On the other hand, with cerebral PMMR imaging, it has been known that T_1_ values correlates significantly with rectal temperature, though T_2_ values did not.^13^ This discrepancy between hepatic and cerebral PMMR imaging suggests organ-specific MR relaxation times after death.

Our study has four limitations. One is that we could not examine 1.5T MR imaging data of healthy volunteers, because an approximately 2-minute breath hold is necessary to obtain comparative imaging data. T_1_ and T_2_ values depend on the type of MR imaging unit and scan conditions; thus may differ on different imaging units or with different parameters.^23^ In our study, we had to compare hepatic PMMR imaging data we generated with published data from clinical MR imaging of the liver in the literature. The second limitation is that the standard deviation of T_1_ value of our subjects were large compared to that of living subjects as reported by de Bazelaire et al.^22^ Decomposition of the liver progresses in cases in which a long period of time elapsed after death. In our study, PMMR imaging was performed 7–96 hours after the confirmation of death. Our subjects had been kept in cold storage, which reduces decomposition of the body. Nevertheless, the wide deviation in the time after death may have caused differences of the degree of decomposition in each body, thereby resulting in increased standard deviation values. Also, a possible uneven rate of decomposition within the liver of a deceased subject may cause a difference in T_1_ shortening effects between the right anterior and left lateral segments of the liver. The third limitation is that B_1_ inhomogeneity correction was not performed with our relaxation time map creation tool, which is considered to have resulted in regional signal variation. A greater variation can be induced in the T_1_ map which is based on gradient echo sequences than the T_2_ map which is based on spin echo sequences. The fourth limitation is that we did not directly measure the pH, fat, water, and paramagnetic substances. Such measurement with an MR imaging system^46–48^ would enable estimation of the level of effect of relaxation time changes of each factor on the total liver relaxation time.

## Conclusion

In conclusion, with hepatic PMMR imaging, T_1_ values did not significantly correlate with the rectal temperature, but T_2_ values did correlate. Postmortem interval had no effect on T_1_ and T_2_ values on hepatic PMMR imaging. Reduction in body temperature after death is considered to induce T_1_ and T_2_ value changes on hepatic PMMR imaging.

## Figures and Tables

**Table 1.** Scan parameters of postmortem magnetic resonance (PMMR) imaging based on the syngo MapIt method

Scan parameters	T_1_ map (GE)	T_2_ map (SE)
TR/TE (ms)	15/1.62	2000/30, 60, 90, 120, 150
Flip angle	5°, 26°	180°
Slice thickness/gap (mm)	3/0	5/1
Matrix size (mm)	1.2 × 1.2	1.2 × 1.2
Field of view (FOV) (mm)	300	300
Scan time (min)	2.1	3.6
Number of slices	22 (3D)	11

GE, gradient echo; SE, spin echo; TR, repetition time; TE, echo time; 3D, three-dimensional

**Table 2.** T_1_ and T_2_ values (ms) in the anterior segment of the right lobe and the lateral segment of the left lobe on hepatic postmortem magnetic resonance imaging

	T_1_ value (ms)	T_2_ value (ms)
Anterior segment of the right lobe	524 ± 112	42 ± 6
Lateral segment of the left lobe	472 ± 104	43 ± 8

No significant difference is found between the two sites. (T_1_: *P* = 0.123, T_2_: *P* = 0.823).
